# Clinical, Radiological and Ultrasonographic Findings Related to Knee Pain in Osteoarthritis

**DOI:** 10.1371/journal.pone.0092901

**Published:** 2014-03-27

**Authors:** Keith K. W. Chan, Regina W. S. Sit, Ricky W. K. Wu, Allen H. Y. Ngai

**Affiliations:** 1 School of Public Health and Primary Care, Faculty of Medicine, The Chinese University of Hong Kong, Hong Kong; 2 Jockey Club School of Public Health and Primary Care, Faculty of Medicine, The Chinese University of Hong Kong, Hong Kong; 3 The Hong Kong Institute of Musculoskeletal Medicine, Hong Kong; University of Maryland, College Park, United States of America

## Abstract

**Background:**

Pain is the predominant symptom of knee osteoarthritis (OA) and the main reason of disability. Ultrasound is now one of the new imaging modality in Musculoskeletal medicine and its role in assessing the pain severity in the knee osteoarthritis is evaluated in this study.

**Objectives:**

(1) To study the correlation between ultrasonographic (US) findings and pain score and (2) whether ultrasonographic findings show a better association of pain level than conventional X-rays in patients suffering from primary knee osteoarthritis.

**Methods:**

In this multi-center study, 193 patients with primary knee OA were asked to score their average knee pain using the Western Ontario and McMaster Universities Arthritis (WOMAC) questionnaire;patients would then go for a radiological and an US evaluation of their painful knee. Findings from both imaging modalities will be studied with the associated pain score.

**Results:**

Ultrasound showed that knee effusion has positive correlation with pain score upon walking (r = 0.217) and stair climbing (r = 0.194). Presence of suprapatellar synovitis had higher pain score on sitting (Spearman's Rank correlation  = 0.355). The medial(r = 0.170) and lateral meniscus protrusion (r = 0.201) were associated with pain score upon stair climbing.

**Conclusions:**

Our study found that both imaging modalities shown some significant association with the aspect of pain; neither one is clearly better but rather complementary to each other. A trend is found in both modalities: walking pain is related to pathologies of the either the lateral or medial tibiofemoral joint(TFJ)while stair climbing pain is related to both tibiofemoral joint pathologies and also to the patellofemoral joint (PFJ) pathology. This suggested that biomechanical derangement is an important aspect in OA knee pain.

## Introduction

Osteoarthritis (OA) is one of the most common medical conditions in elderly people.[1] Worldwide estimates suggested that 9.6% of men and 18.0% of women aged 60 years have symptomatic OA of the hips or knees.[2] It is also the most common reason for restricted daily activity [3] with significantly impact on the quality of life among affected people.[4]

Pain is the predominant symptom of OA knee and is the main reason for medical consultation; besides, it the main reason of disability especially during painful episodes.[5]–[7].

Conventional X-rays were used traditionally in diagnosing OA knee by demonstrating the presence of osteophytes, bone deformities and joint space narrowing. However, studies on OA knee had demonstrated that there was not much correlation between the X-rays findings and the severity of OA knee pain.[8]–[12] Recent concept on osteoarthritis is that OA knee is a whole joint disease involving not only the bones and cartilages; but also involves pathologies in the intra and periarticular soft tissues.[13] One possible reason for the weak correlation is that while conventional X-rays is good at picking up bony lesions, they are falling short in giving accounts on the whole joint by its inability to directly visualize articular cartilage, synovial recesses, menisci and other soft tissues involved in the pathophysiology of OA knee.

Ultrasound (US) imaging is a non-invasive, widely available and relatively inexpensive technique that can be used to image soft tissues. With the advancement of technology, the newer US machine models can be equipped with high frequency probes that are very useful for musculoskeletal imaging to study the periarticular and intraarticular structures.[14]–[17] A study on OA knee had showed that US findings of knee effusion and protrusion of medial meniscus with displacement of the medial collateral ligament are associated with pain in OA knee.[18] Clinicians can now use musculoskeletal US to detect soft tissue pathologies in OA knee to supplement clinical and/or radiological diagnoses.

This study looks at the intraarticular and peri-articular soft tissue changes found among OA knee patients using real-time musculoskeletal US imaging. We aim to investigate (1) the probable correlation between ultrasonographic findings and pain score and (2) whether ultrasonographic findings show a better association of pain level than conventional X-rays in patients suffering from primary knee osteoarthritis. The pain score under the section of Western Ontario and McMaster Universities Arthritis (WOMAC) Index will be used in this study.[19]

## The Study

### Setting and patients

The study is a multicenter study undertaken in Hong Kong at the primary health care level in three different clinical settings: two private general practice clinics, two public primary care outpatient clinics and one primary care clinic of a private hospital. These three settings cover most of the primary health care consultations in Hong Kong.

Convenient sampling was used in this study. Adult patients over 40 years of age attending one of the five primary health care clinics were asked for presence of knee pain in one or both knees for the more than one month. Those patients with a positive answer were screened for presence of OA knee using the ACR clinical criteria of OA knee.[20] (Appendix S1) All patients identified to have OA knees were included in the study. Patients were excluded if he or she (1) had a gouty or pseudo-gouty attack over the knee with the week prior the study; (2) had direct trauma to the knee within the past month prior to the study; (3) had intra-articular injection or aspiration within the month prior to the study; (4) had previous knee operation; (5) declined to undergo either the US or X-ray knee examinations or (6) declined to be included in the study.

## Methods

Ethics approval had been sought and endorsed from the Kowloon West Cluster, Clinical Research Ethics Committee (KWC-CRE), Hong Kong. Written consent was obtained from patients who agreed to participate in the study; the content of the consent form was approved by the ethics committee.

Patients were asked to score their average knee pain during the past month as a baseline on a 10 cm visual analogue scale (VAS) from 0 to 100 mm. If both knees were painful, the most symptomatic knee was used for study. (Appendix S2)

Demographic data obtained from patient included age, sex, height and weight, and body mass index. Patients would then self-complete the pain subscale section of the Chinese version of the WOMAC questionnaire. (Appendix S3) A nurse was available for help if patients needed assistance. Patients would then go for a radiological and an US evaluation of their more painful knee.

### Radiological Evaluation

All patients would have X-rays of their more painful knee. A standing weight bearing anterior-posterior (AP)[21] view was used to assess the medial and the lateral tibial femoral joints (TFJs) and a skyline view of the knee to assess the patello-femoral joint (PFJ). The X-rays were then assessed independently using a standardized scoring sheet and also be graded according to the Kellgren and Lawrence (K/L) method of classification[22] from grades 0 to 4 by two fellow radiologists blinded to the clinical and US results and to each other. (Appendix S4)

### Ultrasound Evaluation

All patients then underwent an US examination on the same knee using a standardized protocol with Esoate (MyLab 25 model) with a linear probe (model LA435 12–18 Mhz) and a centre frequency 13 MHz. (Appendix S5) Gray-scale were used during the screening procedure. Power Colour Doppler was used to check for blood flow in the synovium. To avoid examiner bias, all US examiners had to attend, prior to the study, a standardization workshop under the guidance of an expert specialized in musculoskeletal US scanning. All ultrasound examiners were blinded from the X-ray results during the ultrasound examination and all relevant ultrasound findings as stated in the protocol were saved for an independent reassessment later if necessary by a second examiner.

### Statistical analysis

Statistical analyses were performed using the statistical package PASW Statistics 18. Pearson's correlation and Spearman's Rank correlation were used to examine the association among pain scores, ultrasound and X-ray findings where appropriate. Difference of pain scores in X-ray grading as well as trend were examined by Analysis of variance (ANOVA). Two tailed p-values at level of 0.05 or less were considered to be of statistical significance.

## Results

A total of 193 patients were recruited in this study. Table 1 summarized the demographic data as well as the characteristics of all subjects with pain scores, ultrasound and X-ray findings. Patients answered zero mark in pain score were assigned as no pain in such aspect. The distribution of the five pain scores in relation to walking, stair climbing, lying, sitting and standing were studied. Comparison of pain scores on walking and stair climbing with different ultrasound and X-ray findings were presented in Table 2 with significant figures highlighted. Other significant findings not shown in these 2 tables include (1) presence of suprapatellar synovitis had a moderate strong correlation with higher pain score on sitting (Spearman's Rank correlation  = 0.355); (2) knee effusion has positive correlation with pain score upon walking (0.217) and climbing (0.194) but not with sitting; (3) the narrower the minimal joint width of the medial TFJ (r = −0.175) and the wider the joint width at the lateral point of the lateral TFJ (r = 0.236) on the standing X-rays, the stronger the pain score on standing.

**Table 1:** Characteristics of the study subjects (N = 193).

	Women (n = 143)		Men (n = 50)		Total (n = 193)	
Age (year)	58.9	±13.5	59.3	±15.1	59.0	±13.9
Weight (kg)	60.4	±10.0	70.4	±10.1	63.0	±10.9
Height (m)	1.56	±0.06	1.67	±0.05	1.59	±0.08
BMI (kg/m^2^)	24.9	±4.1	25.2	±3.1	25.0	±3.9
**Pain score (non-zero)**					n	mean± sd
Walking					154	4.1±2.3
Stair climbing					186	5.6±2.5
Lying					97	3.5±2.6
Sitting					87	2.6±2.2
Standing					130	3.4±2.2
**Ultrasound (Front)**						
Patient supine with knee flexed to 30^o^						
Knee effusion in supra-patella recess (mm)					125	6.73±4.36
Supra-patella Synovitis, synovial thickness (mm)					73	4.36±2.21
Sky view with patient supine and knee fully flexed, cartilage thickness at						
Lateral femoral condyle (maximal thickness, mm)					193	2.35±0.67
Lateral femoral condyle (minimal thickness, mm)					193	1.31±0.58
Medial femoral condyle (maximal thickness, mm)					193	2.28±0.76
Medial femoral condyle (minimal thickness, mm)					193	1.15±0.61
Ultrasound (Medial) - Patient supine with external rotation of leg and knee flexed to 10^o^						
Medical meniscus extrusion(mm)					144	4.57±2.33
Maximum length of osteophyte from level of cortical bone(mm)					152	3.77±2.27
Ultrasound (Lateral) - Patient supine with internal rotation of leg and knee flexed to 10^o^						
Lateral meniscus extrusion (mm)					137	4.01±1.85
Maximum length of osteophyte from cortical bone (mm)					149	3.19±1.73
**X-ray**						
Joint width of the medial TFJ compartment						
Minimal joint					193	2.1±1.5
Medial point					193	8.5±2.9
Mid-point					193	4.6±1.9
Joint width of the lateral TFJ compartment						
Minimal joint					193	1.4±1.1
Medial point					193	11.3±3.9
Mid-point					193	4.9±1.6
Joint width of the PFJ at						
Medial facet					193	3.7±1.5
Lateral facet					193	3.0±1.4
KL grading					Grade	n(%)
					0	42(21.8%)
					1	33(17.1%)
					2	43(22.3%)
					3	43(22.3%)
					4	32(16.6%)

**Table 2:** Association among pain score (non-zero) on Walking and Stair Climbing, Ultrasound and X-Ray readings.

Ultrasound readings						
	**Front**					
	Knee effusion in supra-patella recess	Supra-patella Synovitis	Lateral femoral condyle cartilage		Medical femoral condyle cartilage	
			Maximal thickness	Minimal thickness	Maximal thickness	Minimal thickness
Walking	**0.217** *	0.151	−0.024	**−0.181** *	−0.051	−0.143
Stair Climbing	**0.194** *	0.107	0.055	−0.060	0.005	−0.131
	**Medial**		**Lateral**			
	Meniscus extrusion	Max length of osteophyte from level of cortical bone	Meniscus extrusion	Max length of osteophyte from cortical bone		
Walking	0.177	0.136	**0.217** *	0.171		
Stair Climbing	**0.170** *	**0.162** *	**0.201** *	0.127		

X-Ray readings						
	Joint width of the medial TFJ compartment			Joint width of the lateral TFJ compartment		
	Minimal joint	Medial point	Mid-point	Minimal joint	Lateral point	Mid-point
Walking	−0.146	−0.093	**−0.192** *	−0.068	−0.009	−0.021
Stair Climbing	**−0.175** *	−0.070	**−0.179** *	0.043	−0.001	**0.149** *
	Joint width of the PFJ					
	Medial facet	Lateral facet				
Walking	−0.087	−0.155				
Stair Climbing	0.010	**−0.166** *				
	Pain score on walking (non-zero)			Pain score on stair climbing (non-zero)		
KL grading on X-rays	N	Mean±SD	p-value of ANOVA	N	Mean±SD	p-value of ANOVA
0	31	3.3±2.5	**0.002**	41	4.9±2.5	**0.001**
1	22	3.3±2.1	(linear trend<**0.001**)	30	5.1±2.6	(linear trend<**0.001**)
2	30	3.9±2.0		42	5.2±2.2	
3	40	4.3±2.4		43	5.7±2.4	
4	31	5.3±1.9		30	7.2±2.0	
KL grading on X-rays			p-value of t-test			p-value of t-test
0–1	53	3.3±2.3	0.001	71	5.0±2.5	0.009
2–4	101	4.5±2.2		115	5.9±2.3	

*P<0.05; Data presented as Pearson correlation coefficient.

## Discussion

In our study, the higher the KL grading was associated with more pain in general. The finding is in line with a recent systematic review that higher grade of osteoarthritis (K/L 3 or above) is a stronger predictor of pain than lower grades (K/L 2 or less).[8] This study is so designed that the patients recruited should include at least 30 patients from each of the 5 K/L gradings (Grades 0–4) so that the results of the present study is more representative of OA knee in general with different grades of severities.

Our study showed that the presence of suprapatellar synovitis had a moderate strong correlation with higher pain score on sitting whereas such correlation was not found upon walking and standing. This could be explained by a previous study conducted by the author that there exist two types of pain among OA knee patients: a mechanical pain and an inflammatory pain. The former, being biomechanical in nature, is associated with joint movements such as walking and stair claiming whereas the latter is caused by flares of joint inflammation.[23] Synovitis is a sign of joint inflammation and its association with pain when sitting and not with walking or stair climbing suggests that the resting OA knee pain during sitting is more likely inflammatory pain. This finding is also consistent with the EULAR study, which showed no correlation between US inflammatory signs and pain intensity during physical activity.[24]

This study showed that knee effusion has positive correlation with pain score upon walking and climbing, but not with sitting. Similar findings are found in other overseas studies.[17], [19], [24] This suggests that the knee effusion among OA knee patients relates more to mechanical rather than inflammatory pain. Knee effusion has been shown to affect knee mechanics and muscle activity during gait in individuals with knee osteoarthritis and therefore can be a cause of the mechanical pain by itself.[25]

The knee joint is a complex joint consisting of a patellofemoral joint (PFJ) articulating the patella with the femoral condyle and two tibiofemoral joints, one medial and one lateral (medial and lateral TFJs), articulating the medial and lateral tibial plateau with the corresponding femoral condyles.Although X-rays measure mainly bony pathologies and ultrasound on soft tissue pathologies, both the X-ray and ultrasound findings in our study showed two trends in common(1) walking pain is related to pathologies of the either the lateral or medial TFJ;(2) stair climbing pain is related to both TFJ pathologies and also to the PFJ pathology.

Meniscus subluxation was highly associated with painful OA knee.[26] Our US studies showed that lateral meniscal extrusion was associated with increased pain score on walking. X-ray studies, on the other hand, showed pain on walking was associated with the narrowing of the medial TFJ at mid-point. When stair climbing, pathologies from both the medial and lateral TFJ are implicated. The US findings showed that stair climbing pain has a positive correlation to both medial meniscal protrusion and lateral meniscus protrusion; whereas on X-rays, the narrower the medial TFJ at midpoint and the wider the lateral TFJ at midpoint,more the pain. Both of these X-rays findings are commonly found among bow knees (Genu Varus) which are commonly found among OA knee patients.

Pain with stair climbing, especially on descending stairs is a well known feature with OA knee involving the PFJs.[27] Our X-ray studies found that the narrower the lateral facet joint, the more the pain on stair climbing. However, our US finding showed that the thickness of the lateral femoral condyle cartilage, which is a measurement of the lateral PFJ, has no association with stair climbing. Such finding is expected because ultrasound can only measure, through the sky view, the cartilage of the upper part of the femoral condyle. Level walking involves only 15^o^ to 20^o^ of flexion of the femoral condyle during which the lower part of the patella facet is in opposition with the upper part of the femoral condyle,[28] and it is where the ultrasound is measuring. Stair climbing involved 30^o^ to 60^o^ of flexion which is beyond the US measurements.

Some limitations of our study should be mentioned. Although all the ultrasounds were performed by musculoskeletal physicians who are Registrants in Musculoskeletal Ultrasound (RMSK) of the American Registry for Diagnostic Medical Sonography and that all of them had undergone a standardization workshop prior to the study, the US inter-observer reliability was not studied. Recent studies had revealed that there still can be high inter-observer reliability.[29], [30] Secondly, as there is still no widely agreed standardized definition of USG findings or widely acquisition protocols on OA knee US screening, we therefore produce definitions of pathological findings based on some published report.[31]–[34] We employed a conservative “cut off point” for all pathological definitions. This may potentially lead to underestimation of the association of ultrasound finding and pain score. Thirdly, the study design is to recruit equal numbers of OA knee patient from all K/L grading. Such inclusion of mild cases of OA may undermine the pain association and hence may account for the overall low association score.

## Conclusions

Although X-rays and musculoskeletal ultrasound measure different structural tissues of the osteoarthritic knee, our study found that both imaging modalities shown some significant association with the aspect of pain. However, neither one is clearly better, but they rather complementary to each other. Walking pain is related to pathologies of the either the lateral or medial TFJ. Stair climbing pain is related to both TFJ pathologies and also to the PFJ pathology. This suggests that biomechanical derangement is an important aspect that is often overlooked, but needed to be addressed by physicians in the management of OA knee pain. Future research concerning the prognostic value of the different findings from both modalities in planning treatment or intervention can be conducted in order to improve clinical outcomes of patients suffering chronic OA knee.

## Supporting Information

Appendix S1 (DOC)

Appendix S2 (DOC)

Appendix S3 (PDF)

Appendix S4 (DOC)

Appendix S5 (DOC)
