# Correction: Clinical, Radiological and Ultrasonographic Findings Related to Knee Pain in Osteoarthritis

**DOI:** 10.1371/journal.pone.0102268

**Published:** 2014-07-02

**Authors:** The PLOS ONE Staff

The first two authors, Keith K.W. Chan and Regina W.S. Sit, should be noted as contributing equally to this work.
