# Supplementary material for: Clinical, Radiological and Ultrasonographic Findings Related to Knee Pain in Osteoarthritis
Source: PLoS One. 2014 Mar 27;9(3):e92901. doi: 10.1371/journal.pone.0092901 (PMC3968041; doi:10.1371/journal.pone.0092901)
Supplement: Appendix S1 — (DOC) [file pone.0092901.s001.doc]

American College of Rheumatology: Criteria of Osteoarthritis of the Knee using history and clinical examination[[1]](#endnote-2).

1. Pain in the knee for more than 1 month (Must) □

*And 3 of the following*

1. over 50 years old □
2. less than 30 minutes of morning stiffness □
3. Crepitus on active motion □
4. Bony tenderness □
5. Bony enlargement □
6. No palpable warmth of synovium □

1. Altman R, Asch E, Bloch D, et al. “Development of Criteria for The Classification And Reporting of Osteoarthritis, Classification of Osteoarthritis of The Knee.” Arthritis And Rheumatism 1986; 29:1039. [↑](#endnote-ref-2)
