# Supplementary material for: Clinical, Radiological and Ultrasonographic Findings Related to Knee Pain in Osteoarthritis
Source: PLoS One. 2014 Mar 27;9(3):e92901. doi: 10.1371/journal.pone.0092901 (PMC3968041; doi:10.1371/journal.pone.0092901)
Supplement: Appendix S2 — (DOC) [file pone.0092901.s002.doc]

*DEMOGRAPHIC DATA*

1. Age: __________

2. Sex: Female □^1^ Male □^2^

3. Weight: __________ kg

4. Height: __________ cm

5. BMI: __________

*PAIN ASSESSMENT*

6. How long do you have knee pain?

你有膝關節疼痛多久？ _____ years(年) _____months(月)

7. How would you grade your knee pain over the past month?

請圈出你過去一個月膝關節疼痛的程度。

0 1 2 3 5 6 7 8 9 10

No pain Pain as bad

as it could be

無痛 極痛苦

8. Do you have an increase of pain over the past 2 days?

你過去兩天膝關節的疼痛有沒有增加？

No, go to end. 沒有：本問卷完結。

Yes, go to question 9. 有：請回答問題九。

9. How would you grade your knee pain over the past 2 days?

請圈出你過去兩天膝關節疼痛的程度。

0 1 2 3 5 6 7 8 9 10

No pain Pain as bad

as it could be

無痛 極痛苦
