# Supplementary material for: Clinical, Radiological and Ultrasonographic Findings Related to Knee Pain in Osteoarthritis
Source: PLoS One. 2014 Mar 27;9(3):e92901. doi: 10.1371/journal.pone.0092901 (PMC3968041; doi:10.1371/journal.pone.0092901)
Supplement: Appendix S3 — (PDF) [file pone.0092901.s003.pdf]

WOMAC VA3.1 QUESTIONNAIRE WOMA

A部

**疼 痛**

請回想在過去48小時您因為關節炎而引致 (接受研究的關節)感到的痛楚。

（請用「🗴」號標出答案。）

| 問題：　您感覺有多痛？   1. 在平地上步行。   不痛 極痛 |  | 只限研究人員使用  PAIN1 |
| --- | --- | --- |
| 1. 上落樓梯。   不痛 極痛 |  | PAIN2 |
| 1. 晚上躺在床上時，如，　干擾睡眠的疼痛。   不痛 極痛 |  | PAIN3 |
| 1. 坐著或躺著的時候。   不痛 極痛 |  | PAIN4 |
| 1. 直立的時候。   不痛 極痛 |  | PAIN5 |

Copyright©1996 Nicholas Bellamy

All Rights Reserved

VB – Hong Kong

WOMAC VA3.1 QUESTIONNAIRE WOMB

B部

**僵硬的感覺**

請回想在過去48小時您因為關節炎而引致 (接受研究的關節)僵硬(不是指疼痛)的感覺。

僵硬是指關節活動時靈活性減低的感覺。

（請用「🗴」號標出答案。）

| 1. 早上一醒來時你的僵硬感覺有多嚴重?   不覺 極度  僵硬 僵硬 |  | 只限研究人員使用  STIFF6 |
| --- | --- | --- |
| 1. 日間經過坐著，躺著，或休息後，您僵硬的感覺有多嚴重？   不覺 極度  僵硬 僵硬 |  | STIFF7 |

Copyright©1996 Nicholas Bellamy

All Rights Reserved

VB – Hong Kong

WOMAC VA3.1 QUESTIONNAIRE WOM_ｃ１－３_

Ｃ部

**進行日常活動的困難**

請回想在過去48小時，您因為 　　(接受研究的關節)患有關節炎而令您在進行日常體力活動時的困難。

這里的意思是指你在**行動上及照顧自己**的能力。

（請用「🗴」號標出答案。）

| 1. 問題：　請問您的困難程度是什麼?   沒有 極度  困難 困難 |  | 只限研究人員使用  PFTN8 |
| --- | --- | --- |
| 1. 上樓梯。   沒有 極度  困難 困難 |  | PFTN9 |
| 1. 從坐時站起來。   沒有 極度  困難 困難 |  | PFTN10 |
| 1. 站立。   沒有 極度  困難 困難 |  | PFTN11 |
| 1. 彎身向地下。   沒有 極度  困難 困難 |  | PFTN12 |
| 1. 在平地上步行。   沒有 極度  困難 困難 |  | PFTN13 |

Copyright©1996 Nicholas Bellamy

All Rights Reserved

VB – Hong Kong

WOMAC VA3.1 QUESTIONNAIRE WOM_ｃ2－３_

Ｃ部

**進行日常活動的困難**

請回想在過去48小時，您因為 　　(接受研究的關節)患有關節炎而令您在進行日常體力活動時的困難。

這里的意思是指你在**行動上及照顧自己**的能力。

（請用「🗴」號標出答案。）

| 問題：　請問您的困難程度是什麼?   1. 上落車，或上落巴士。   沒有 極度  困難 困難 |  | 只限研究人員使用  PFTN14 |
| --- | --- | --- |
| 1. 行街買東西。   沒有 極度  困難 困難 |  | PFTN15 |
| 1. 穿上短襪或長襪。   沒有 極度  困難 困難 |  | PFTN16 |
| 1. 起床。   沒有 極度  困難 困難 |  | PFTN17 |
| 1. 脫下短襪或長襪。   沒有 極度  困難 困難 |  | PFTN18 |
| 1. 挨在床上   沒有 極度  困難 困難 |  | PFTN19 |

Copyright©1996 Nicholas Bellamy

All Rights Reserved

VB – Hong Kong

WOMAC VA3.1 QUESTIONNAIRE 　WOM_ｃ3－３_

Ｃ部

**進行日常活動的困難**

請回想在過去48小時，您因為 　　(接受研究的關節)患有關節炎而令您在進行日常體力活動時的困難。

這里的意思是指你在**行動上及照顧自己**的能力。

（請用「🗴」號標出答案。）

| 問題：　請問您的困難程度是什麼?   1. 踏進或踏出浴缸。   沒有 極度  困難 困難 |  | 只限研究人員使用  PFTN20 |
| --- | --- | --- |
| 1. 坐著。   沒有 極度  困難 困難 |  | PFTN21 |
| 1. 坐上座廁或離來座廁。   沒有 極度  困難 困難 |  | PFTN22 |
| 1. 做粗重家務。   沒有 極度  困難 困難 |  | PFTN23 |
| 1. 做輕巧家務。   沒有 極度  困難 困難 |  | PFTN24 |

Copyright©1996 Nicholas Bellamy

All Rights Reserved

VB – Hong Kong

**WOMAC OSTEOARTHRITIS INDEX VERSION VA3.1**

| 病人需知  A,B及C部的問題是用以下的形式來發問。請在橫線上劃上  「🗴」號來作答。  舉例：   1. 如把「🗴」號劃在下列橫線的左端，即表示您不痛。   🗴  不痛 極痛   1. 如把「🗴」號劃在下列橫線的右端，即表示您感到極痛。   🗴  不痛 極痛   1. 請注意： 2. 如「🗴」號的位置越靠近右端，即表示越覺疼痛。 3. 如「🗴」號的位置越靠近左端，即表示疼痛越少。 4. 勿把「🗴」號劃在線的兩端以外。   請用上述的尺度方法來說明您在過去48小時所感受到的疼痛、僵硬及行動不便程度。  回答問卷時，請以您的 (接受研究的關節)來作考慮。  請指出您認為由於 (接受研究的關節)患有關節炎而引起的疼痛、僵硬及行動不便的嚴重程度。  您接受研究的關節是由專業健康護理人員所選定。如不確定那個是您接受研究的關節，請在填寫問卷前查詢。 |
| --- |

Copyright©1996 Nicholas Bellamy

All Rights Reserved

VB – Hong Kong
