# Supplementary material for: Clinical, Radiological and Ultrasonographic Findings Related to Knee Pain in Osteoarthritis
Source: PLoS One. 2014 Mar 27;9(3):e92901. doi: 10.1371/journal.pone.0092901 (PMC3968041; doi:10.1371/journal.pone.0092901)
Supplement: Appendix S4 — (DOC) [file pone.0092901.s004.doc]

*PROTOCOL USED*

Two views were taken from the knee under studied.

- The standing antero-posterior (AP) view of the knee in the weight bearing position was employed in this study to assess the medial and the lateral tibiofemoral joints (TFJs). In this standing AP protocol, the patient was positioned with the knees as fully extended as possible, weight distributed evenly between both legs, and the toes straight ahead.[[1]](#endnote-2) (Fig 1)


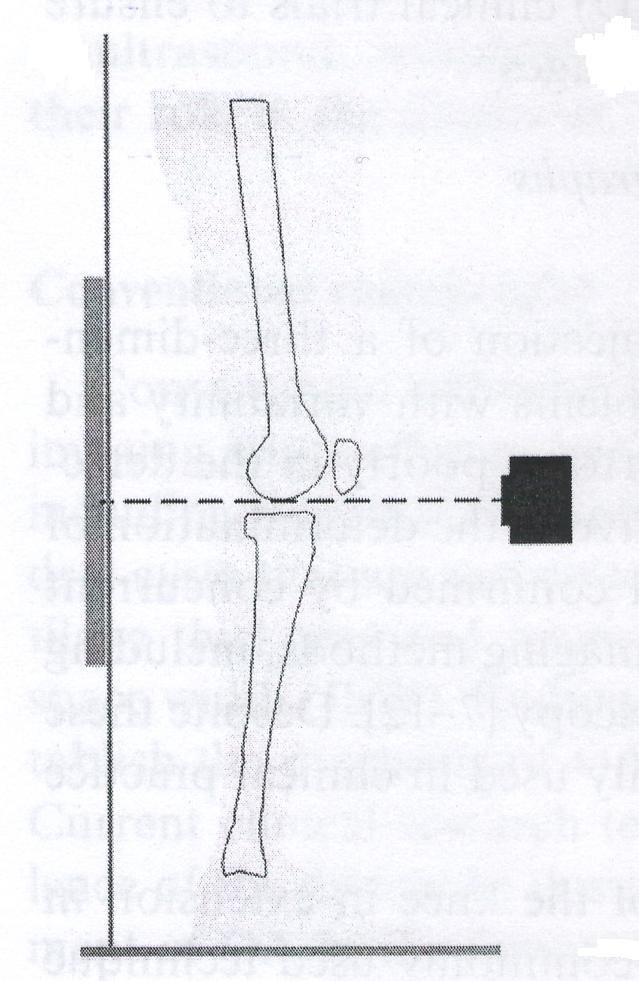


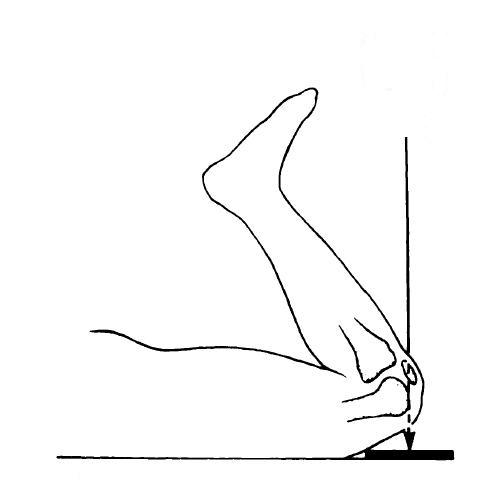


**Figure 1:** *Positioning of the subject in AP view* **Figure 2:** *Skyline view according to Settegast*

*with knee as fully extended as possible*

- The skyline view of the patella was taken using Settegast method in this study and was used to assess the lateral and medial facets of the patello-femoral joint (PFJ).[[2]](#endnote-3) (Figure 2)

*ASSESSMENTS*

| **AP view:** The minimum TFJ space (to the nearest 0.1mm) is measured according to the landmarks recommended by Buckland-Wright[[3]](#endnote-4), by using a 10x magnifying lens (Nakabayashi, Japan) fitted with graticule with 0.1 mm divisions. | | | |
| --- | --- | --- | --- |
| Joint width of the **medial** TFJ compartment (mm) measured at | the site with the minimal joint width | |  |
|  | the most medial point (border) of the compartment | |  |
|  | the mid-point of the compartment | |  |
| Joint width of the **lateral** TFJ compartment (mm) measured at | the site with the minimal joint width | |  |
|  | the most lateral point (border) of the compartment | |  |
|  | the mid-point of the compartment | |  |
| **Skyline view:** The minimum PFJ joint space (to the nearest 0.5mm) is measured with a standard millimeter-graded plastic ruler, from the bright radio-dense band of subchondral cortex on the patella to the articular margin of the femoral trochlea. The PFJ is taken as corresponding to the central 3/4 of the articular surfaces of the femoral trochlea[[4]](#endnote-5). | | | |
| Minimum joint space of **medial** facet of the PFJ (mm) | | |  |
| Minimum joint space of **lateral** facet of the PFJ (mm) | | |  |
| **The Kellgren and Lawrence method of classification[[5]](#endnote-6) was used where Grade**   1. normal, 2. doubtful osteophyte, 3. definite osteophyte, 4. moderate joint space narrowing and 5. severe joint space narrowing. | | | |
| OA knee grading according to  Kellgren and Lawerence (0-4) | |  | |

<<< Joint width was changed into a categorical variable using the median and quartile values of the database analyzed: cut-offs were 2 mm (quartile), 3.5 mm (median), and 5 mm (quartile) for statistical analysis.[[6]](#endnote-7) >>>

1. Guermazi A. et al. Imaging in osteoarthritis. Rheum Dis Clin North Am. 2008 Aug;34(3):645-87. [↑](#endnote-ref-2)
2. Eisenberg RL, Dennis CA, May CR. Radiographic Positioning 2^nd^ Ed. P. 134. Little Brown & Company. [↑](#endnote-ref-3)
3. Buckland-Wright JC. Protocols for precise radio-anatomical positioning of the tibiofemoral and

   patellofemoral compartments of the knee. Osteoarthritis Cartilage 1995;3:71-80. [↑](#endnote-ref-4)
4. Boegard T, Rudling O, Petersson IF, Sanfridsson J, Saxne T, Svensson B, Jonsson K. Joint-space width in the axial view of the patello-femoral joint. Definitions and comparison with MR imaging.
   Acta Radiol. 1998 Jan;39(1):24-31. [↑](#endnote-ref-5)
5. Kellgren JH, Lawrence JS Radiological assessment of osteoarthritis. Ann Rheum Dis 1957; 16: 494-501. [↑](#endnote-ref-6)
6. Gossec L. et al. Comparative evaluation of three semi-quantitative radiographic grading techniques for knee osteoarthritis in terms of validity and reproducibility in 1759 X-rays: report of the OARSI - OMERACT task force. Osteoarthritis and Cartilage (2008) 16, 742e748 [↑](#endnote-ref-7)
