# Supplementary material for: Clinical, Radiological and Ultrasonographic Findings Related to Knee Pain in Osteoarthritis
Source: PLoS One. 2014 Mar 27;9(3):e92901. doi: 10.1371/journal.pone.0092901 (PMC3968041; doi:10.1371/journal.pone.0092901)
Supplement: Appendix S5 — (DOC) [file pone.0092901.s005.doc]

| **Front** | | | | | | | |
| --- | --- | --- | --- | --- | --- | --- | --- |
| Patient supine with knee flexed to 30^o^ | Supra-patella | Not seen | | Normal | | Abnormal | |
|  | Quadriceps tendons (LS &TS) |  | |  | |  | |
|  | Supra-patellar bursa |  | |  | |  | |
|  | Pre-patellar bursa |  | |  | |  | |
|  | Knee effusion in supra-patella recess (mm)* |  | |  | |  | |
|  |  | Absent | | Diffuse | | Nodular | |
|  | Supra-patella Synovitis** (thickness in mm) |  | |  | |  | |
|  |  | Supra-patellar pouch | | Lateral joint margin | | Medial joint margin | |
|  | Blood flow in synovium by colour Flow –Doppler[[1]](#endnote-2) (spot count) |  | |  | |  | |
| * Knee effusion-A compressible anechoic area in the supra-patellar recess with a maximum AP diameter ≥ 4mm at medial sagittal plane^2^  ^**^ Synovitis- hypoechoic synovial hypertrophy of thickness ≥ 4mm with diffuse or nodular appearance at medial sagittal plane[[2]](#endnote-3) | | | | | | | |
| Patient supine with knee flexed to 45^o^ | Infra-patella | Not seen | | Normal | | Abnormal | |
|  | Patella tendon (LS&TS) |  | |  | |  | |
|  | Superficial infrapatellar bursa |  | |  | |  | |
|  | Deep infrapatellar bursa |  | |  | |  | |
| Sky view with patient supine and knee fully flexed | Qualitative study | Loss of normal sharpness of synovial space-cartilage interface | Loss of clarity of the cartilaginous layer | | Narrowing of joint cartilage | | Increase intensity of the posterior cartilage interface |
|  | Articular Cartilage of  the femoral condyles in general[[3]](#endnote-4) |  |  | |  | |  |
|  | Thickness in mm | Maximal thickness | | | Minimal thickness | | |
|  | Lateral femoral condyle (mm) |  | | |  | | |
|  | Medical femoral condyle (mm) |  | | |  | | |
|  | Intercondylar notch (mm) |  | | | | | |
| **Medial** | | | | | | | |
|  |  | Not seen | | Normal | | Abnormal | |
| Patient supine with external rotation of leg and knee flexed to 10^o^ | Medical collateral ligament |  | |  | |  | |
|  | Medial meniscus extrusion^†^ |  | |  | |  | |
|  | Maximum length of osteophyte from level of cortical bone (mm) |  | |  | |  | |
|  | Distension of joint capsule(mm) ^‡^ |  | |  | |  | |
|  | Anserine bursa (LS&TS) |  | |  | |  | |
| ^†^ Extrusion of the medial or lateral meniscus is present if the meniscus is extruded by > 3 mm from the longitudinal joint line[[4]](#endnote-5).  ^‡^Measure indirectly as the longest vertical distance from the inner surface of the medial collateral ligament to level of cortical bone[[5]](#endnote-6) | | | | | | | |
| **Lateral** | | | | | | | |
| Patient supine with internal rotation of leg and knee flexed to 10^o^ |  | Not seen | | Normal | | Abnormal | |
|  | Lateral collateral ligament |  | |  | |  | |
|  | Lateral meniscus extrusion^†^ |  | |  | |  | |
|  | Maximum length of osteophyte from cortical bone (mm) |  | |  | |  | |
|  | Tensor fascia lata |  | |  | |  | |
| **Back** | | | | | | | |
| Patient prone with knee fully extended |  | Not seen | | Normal | | Abnormal | |
|  | Baker cyst (LS&TS)^#^ |  | |  | |  | |
| ^#^ A hypoechogenic collection of fluid in the medial posterior knee that communicates with the joint and is > 4 mm on long and short axes^4^ | | | | | | | |

1. Kristoffersen H et al. Indications of inflammation visualized by ultrasound in osteoarthritis of the knee. Acta Radiol. 2006 Apr;47(3):281-6. [↑](#endnote-ref-2)
2. Agostino et.al. EULAR report on the use of ultrasonography in painful knee osteoarthritis. Part 1: Prevalence of inflammation in osteoarthritis. Annals of the Rheumatic Diseases 2005;64:1703-1709. [↑](#endnote-ref-3)
3. W.Grassi, G.Lamanna, A.Farina, C.Cervini. Sonographic imaging of normal and osteoarthritic cartilage. Seminars in Arthritis and Rheumatism, Volume 28, Issue 6, Pages 398-403 [↑](#endnote-ref-4)
4. de Miguel Mendieta E, Cobo Ibáñez T, Usón Jaeger J, Bonilla Hernán G, Martín Mola E. Clinical and ultrasonographic findings related to knee pain in osteoarthritis. Osteoarthritis Cartilage (2006) June;14(6):540-544 [↑](#endnote-ref-5)
5. Chhem RK, Cardinal E. Guidelines and gamuts in musculoskeletal ultrasound. 1^st^ ed. P. 166, New York, Wiley-Liss, 1999. [↑](#endnote-ref-6)
